# Serum Sulfatide Levels as a Biomarker of Active Glomerular Lesion in Patients with Anti-Neutrophil Cytoplasmic Antibody-Associated Vasculitis: A Single Center Pilot Study

**DOI:** 10.3390/jcm11030762

**Published:** 2022-01-30

**Authors:** Makoto Harada, Takero Nakajima, Yosuke Yamada, Daiki Aomura, Akinori Yamaguchi, Kosuke Sonoda, Naoki Tanaka, Koji Hashimoto, Yuji Kamijo

**Affiliations:** 1 Department of Nephrology, Shinshu University Hospital, Matsumoto 390-8621, Japan; yosukeyama@shinshu-u.ac.jp (Y.Y.); aomura91@gmail.com (D.A.); akinoleader@hotmail.co.jp (A.Y.); kosuke_s@live.jp (K.S.); khashi@shinshu-u.ac.jp (K.H.); 2 Department of Metabolic Regulation, Shinshu University School of Medicine, Matsumoto 390-8621, Japan; nakat@shinshu-u.ac.jp (T.N.); naopi@shinshu-u.ac.jp (N.T.); 3 International Relations Office, Shinshu University School of Medicine, Matsumoto 390-8621, Japan

**Keywords:** serum sulfatides, anti-neutrophil cytoplasmic antibody-associated vasculitis, crescentic glomerulonephritis

## Abstract

Sulfatides are glycosphingolipids that are associated with coagulation and platelet aggregation. Anti-neutrophil cytoplasmic antibody (ANCA)-associated vasculitis (AAV) activates platelet function and often leads to thrombotic complications. These facts suggest an association between serum sulfatides and AAV. We aimed to clarify the significance of serum sulfatide levels in patients with AAV. We conducted a retrospective, single-center, observational pilot study that included 35 patients who developed AAV and 10 control patients who were candidates for living-donor kidney transplantation. We compared serum sulfatide levels between the control and AAV patients. We analyzed the differences in serum sulfatide levels among four classes (focal, crescentic, mixed, and sclerotic class) of glomerular lesions that were categorized by histopathologic classification of ANCA-associated glomerulonephritis. Serum sulfatide levels in patients with AAV were significantly lower than those in the controls. Serum sulfatide levels were significantly different between the four classes. Additionally, serum sulfatide levels in the crescentic class were significantly lower than those in the other classes. Serum sulfatide levels were significantly correlated with albumin, cholesterol, C-reactive protein, and pentraxin 3. In conclusion, serum sulfatide levels are significantly correlated with inflammation, reflecting crescentic glomerulonephritis, which is an active glomerular lesion in AAV patients.

## 1. Introduction

Anti-neutrophil cytoplasmic antibody (ANCA)-associated vasculitis (AAV) is a systemic vasculitis that causes organ injuries, particularly to the kidneys and lungs, making it a life-threatening condition [1,2,3]. Lung injuries present with interstitial pneumonitis and/or alveolar hemorrhage [1,2,3]. In addition, neuritis and/or purpura often develops [1,2,3]. Rapidly progressive glomerulonephritis caused by necrotizing vasculitis in the glomeruli is one of the important kidney lesions that is often detected in patients who develop AAV, leading to end-stage kidney disease [1,2]. Kidney biopsy is a necessary procedure to evaluate these kidney lesions and the disease activity of AAV [4,5]. However, it is impossible to perform kidney biopsy in all patients with AAV [6], because some patients are already being treated with anti-coagulant or anti-platelet agents or are elderly and have a poor performance status. Recently, serum ANCA titer and C-reactive protein have been used as disease activity markers for AAV [2,7]. However, serum ANCA titers do not always reflect the disease activity or severity of AAV in patients in real time [7]. C-reactive protein levels increase in various pathophysiological conditions, such as infections or malignant tumors, besides AAV or other autoimmune diseases [8]. Therefore, biomarkers that reflect the disease activity of AAV, severity of necrotizing crescentic glomerulonephritis, and kidney prognosis are needed. 

3-O-sulfogalactosylceramides (sulfatides) are glycosphingolipids that are composed of ceramide, galactose, and sulfate [9,10,11]. Sulfatides are produced in the liver, secreted from the liver with lipoproteins, and are abundantly found in the brain, kidney, and digestive tract [9]. Sulfatides are also expressed on the platelet surface and are involved in platelet adhesion and aggregation [12,13]. The mechanism is explained by the binding of sulfatides on the platelet surface to P-selectin on adjacent platelets, facilitating the formation of large, stable platelet aggregates. Sulfatides are also found in serum lipoproteins, where they are the major glycosphingolipid component [14]. Previous studies by our group have demonstrated that intravenous injection of sulfatides significantly prolongs the bleeding time in mice, rats, and rabbits [15,16,17]. These results suggest that serum sulfatides in the lipoprotein particles may prevent coagulation/thrombogenesis by interrupting the sulfatides–P-selectin interaction in platelet adhesion and aggregation [18]. Therefore, we had focused on the association between serum sulfatide levels and cardiovascular disease (CVD)-related vascular thrombosis in previous studies [19,20,21]. We reported a significant association between serum sulfatide levels and CVDs in hemodialysis patients who were at high risk of developing CVDs [19].

Previous studies have suggested that AAV activates platelet function, and platelet function-related markers are significantly associated with the disease activity of AAV [22,23]. Among patients with AAV, the risk of developing thrombotic complications is significantly higher in those with systemic vasculitis [24]. In addition, serum anti-sulfatide antibody levels were shown to be significantly higher in patients who developed AAV than in those who did not [25] (however, we did not investigate anti-sulfatide antibodies, and we did not present any data related to anti-sulfatide antibodies in the current study). These findings suggest the possibility of a significant association between serum sulfatide levels and AAV. 

To clarify the clinical importance of serum sulfatide levels in patients with AAV, we conducted a pilot study, and we compared the serum sulfatide levels between controls and patients with AAV and investigated the association between serum sulfatide levels and active kidney lesions such as crescentic necrotizing glomerulonephritis.

## 2. Materials and Methods

This was a retrospective, single-center, observational, exploratory study that investigated the clinical importance of serum sulfatide levels in patients with AAV. We enrolled 39 patients who were newly diagnosed with AAV at the Department of Nephrology, Shinshu University Hospital, between January 2013 and December 2019. Patients younger than 20 years or those who had been treated with immunosuppressive therapy prior to AAV induction therapy were excluded. In addition, patients who were positive for anti-glomerular basement membrane (GBM) antibody in addition to ANCA (myeloperoxidase [MPO] and/or proteinase 3 [PR3]) were excluded because these patients may receive stronger immunosuppressive therapy. Of the 39 patients, 2 were excluded due to anti-GBM antibody positivity, and 2 were excluded due to treatment at other hospitals. Finally, 35 patients were included in the final analysis. Of the 35 patients, 27 underwent a kidney biopsy. We adapted patients who were being investigated as candidates for living-donor kidney transplantation between 2015 and 2020 in Shinshu University Hospital as controls. We used the data collected before kidney transplantation. This study was approved by the institutional review board of the ethical committee of Shinshu University School of Medicine (approval number: 4954) and was conducted in accordance with the principles of the Declaration of Helsinki. The requirement for written informed consent was waived because of the retrospective nature of the study. The data used in this study are available from the corresponding author upon request.

Based on the algorithm suggested by Watts et al. [26], we defined AAV and categorized each patient as having eosinophilic granulomatous with polyangiitis (EGPA), granulomatous polyangiitis (GPA), microscopic polyangiitis (MPA), or unclassifiable types. Diabetes mellitus was defined as a high level of hemoglobin A1c (HbA1c) (>6.5%), an insulin or hypoglycemic agent prescription, and/or a history of diabetes mellitus listed in the patient’s medical records. Hypertension was defined as antihypertensive drug prescription and/or a history of hypertension, as described in the medical records. End-stage kidney disease was defined as the requirement for maintenance renal replacement therapy or kidney transplantation. Interstitial lung lesions were defined as bilateral interstitial lesions on computed tomographic images. Alveolar hemorrhage was defined as hemoptysis and lung abnormalities on computed tomography that corresponded to hemorrhage. Nerve disorder was defined as neurological symptoms such as numbness and muscle weakness. Thrombotic complications were defined as arterial and/or venous thrombosis detected by ultrasonography, enhanced computed tomography, and/or angiography. 

We measured serum sulfatide levels using a matrix-assisted laser desorption ionization-time of flight mass spectrometry (MALDI-TOF MS) system as in our previous study [27], with a slight modification. Briefly, 50 µL of sera from individuals were mixed with 18 volumes of n-hexane/isopropanol solution (3:2, *v*/*v*) as described previously [27], and total lipids were extracted. For standard quantification of sulfatides, pooled normal human sera (#12181201, lot#BJ10633A, Cosmobio, Tokyo, Japan) were used, and total lipids were extracted in the same manner as above. Prior to this study, we had determined the sulfatide concentration in the pooled human sera. After this, the lipid extracts from the samples and the standard sera were treated with methanolic sodium hydroxide, while being heated, to convert sulfatides to their corresponding lysosulfatides (LS: sulfatides without fatty acids). The LS samples were purified using Mono-tip C18 cartridges (GL Sciences, Tokyo, Japan), and equal amounts of a calibrator N-acetylated LS-sphinganine (LS-d18:0-NAc) were added to each sample. After drying, the LS samples were dissolved in 9-aminoacridine matrix solution (5 mg/mL in 80% methanol; #92817, Merck, Darmstadt, Germany), and 1 µL of the samples were spotted on a MALDI-TOF MS plate. MALDI-TOF MS analysis of LS molecules was performed using a TOF/TOF 5800 system (AB Sciex, Framingham, MA, USA) in negative ion reflector mode with 2-point external calibration using the calibrator LS-d18:0-NAc ([M–H]^-^ 584.310) and LS-(4E)-sphinganine (d18:1) ([M–H]^-^ 540.284) peaks. As in the previous study, we detected seven LS species in normal human sera; among these, LS-sphingadienine (d18:2), d18:1, and phytosphingosine (t18:0) were the major species, comprising more than 80% of the LS species [27]. Thus, we focused on these three LS species in this study. The concentrations of LS-d18:2, d18:1, and t18:0 in each sample were calculated using the data of the standard sera, and the sum of these three was defined as the concentration of serum sulfatides. For each serum, LS samples were prepared in duplicate or triplicate, and at least two spots were analyzed for each replicate in MS. Soluble thrombomodulin (a marker of vascular endothelial injury) was evaluated using the chemiluminescent enzyme immunoassay method (LSI Medience, Corporation, Tokyo, Japan) [28]. Pentraxin 3 (also a marker of vascular endothelial injury and inflammation) was evaluated using the enzyme immunoassay method (LSI Medience, Corporation, Tokyo, Japan) [29]. The estimated glomerular filtration rate was calculated using a previously reported formula [30]. MPO- and PR3-ANCA titers were measured using a chemiluminescence enzyme immunoassay (CLEIA). Blood and urine samples were obtained at the time of hospital admission.

To evaluate the severity and activity of glomerulonephritis in patients who developed AAV, we assessed histopathological findings using a previously suggested histopathologic classification of ANCA-associated glomerulonephritis [4]. Based on the algorithm of this classification, we divided kidney lesions into four groups as follows: biopsies that contained more than 50% of glomeruli with global sclerosis were categorized as the sclerotic class; biopsies that contained more than 50% of normal glomeruli were categorized as the focal class; biopsies that contained more than 50% of glomeruli with cellular and fibro-cellular crescent were categorized as the crescentic class; and those without the features in the previous three categories were categorized as the mixed class. Evaluation was performed by two pathologists who were blinded to the patients’ backgrounds. This classification reflects kidney prognosis [4,5,31], and a previous report from Japan indicated that kidney prognosis in the crescentic class is poorer than in other classes, such as the focal and mixed classes [32]. Patients in the crescentic class have active lesions of ANCA-associated glomerulonephritis, and these patients have a high risk of poor kidney prognosis. Therefore, it is believed that patients categorized in the crescentic class will be treated with intensive immunosuppressive therapy against AAV. Concerning tubulointerstitial lesions in AAV patients, we evaluated the percentage of interstitial fibrosis and tubular atrophy (IFTA) area using two pathologists who were blinded to the patients’ background. Then, we compared the serum sulfatide levels between patients who had IFTA > 25% and those with IFTA < 25%.

The maximum dose of prednisolone (PSL) was adjusted according to the ideal body weight. Methylprednisolone pulse therapy consisted of 500–1000 mg/day of intravenous methylprednisolone administered for three consecutive days. Cyclophosphamide was administered intravenously. The severity of AAV was evaluated based on the Birmingham Vasculitis Score 3 (BVAS-3) at the time of hospital admission [33]. AAV treatment was performed according to the Japanese guidelines for ANCA-positive rapid progressive glomerulonephritis [34].

All continuous variables were evaluated by the Shapiro–Wilk test for normality. The continuous variables exhibiting a normal distribution are presented as means and standard deviations, and those exhibiting a non-normal distribution are presented as medians and interquartile ranges. Categorical variables are presented as numbers (*n*) and percentages (%). Comparisons of continuous variables between two groups were conducted by Student’s *t*-test or Mann–Whitney U test according to whether the variables exhibited a normal or non-normal distribution, respectively. Categorical variables were compared between two groups by Fisher’s exact probability test. Comparisons of clinical parameters between the four histopathologic classes of ANCA-associated glomerulonephritis were conducted by a one-way ANOVA or Kruskal–Wallis test according to whether they followed a normal or non-normal distribution, respectively. We then conducted post hoc comparisons of the clinical parameters of the crescentic class with those of the other classes. In particular, because AAV affects inflammation, coagulation disorders, vascular endothelial injury, and kidney function [1,2], we focused on serum sulfatide levels as well as C-reactive protein, fibrin/fibrinogen degradation products (FDP) D-dimer, soluble thrombomodulin, and estimated glomerular filtration rate (eGFR). The correlations between possible AAV markers (serum sulfatides, C-reactive protein, FDP D-dimer, eGFR, and soluble thrombomodulin) and glomerular lesions, such as the percentage of active glomerular crescent and global sclerosis, were analyzed by Pearson’s correlation tests or Spearman’s rank correlation coefficients according to whether the variable exhibited a normal or non-normal distribution, respectively. The associations between the above possible AAV markers and IFTA > 25% were analyzed using univariate logistic regression analyses. In addition, the correlations between serum sulfatide levels and clinical parameters were also analyzed using Pearson’s correlation tests or Spearman’s rank correlation coefficients according to whether the variable had a normal or non-normal distribution, respectively. The association between clinical outcomes/complications and serum sulfatide levels was evaluated using univariate logistic regression analyses. Statistical significance was set at *p* < 0.05. Analyses were performed using EZR (Saitama Medical Center, Jichi Medical University, Saitama, Japan), which is a graphical user interface for R (The R Foundation for Statistical Computing, Vienna, Austria) [35].

## 3. Results

### 3.1. Comparison of Serum Sulfatide Levels and Clinical Characteristics between Control and AAV Patients

**Figure 1:**
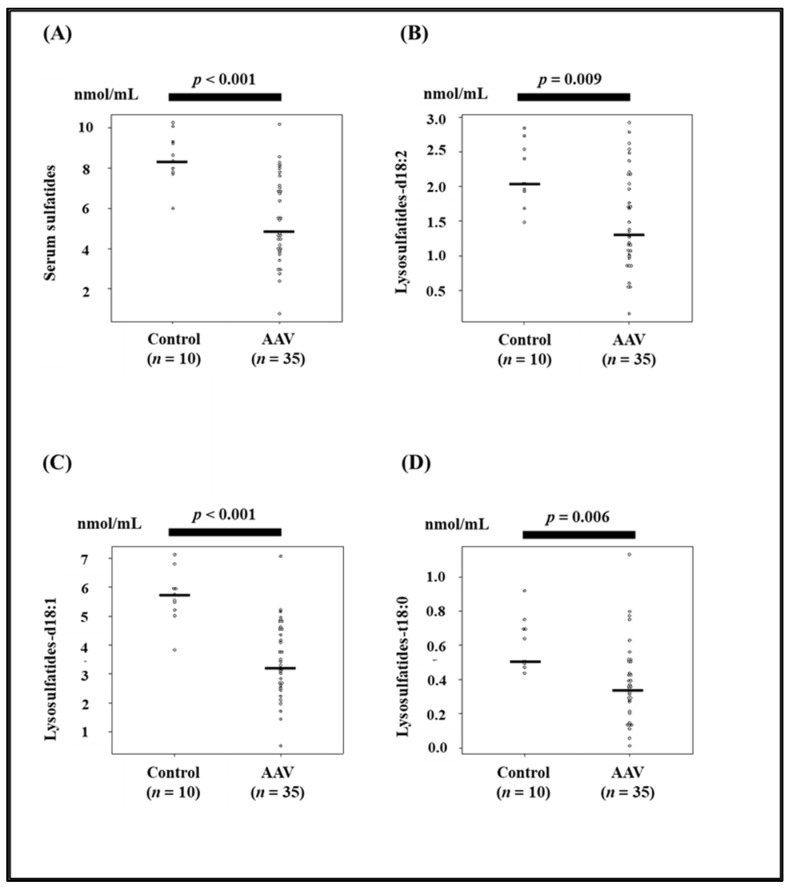
Comparison of the serum sulfatide levels between control and AAV patients. (**A**–**D**) The levels of serum sulfatides and their components (Lysosulfatide-d18:2, Lysosulfatide-d18:1, and Lysosulfatide-t18:0) were significantly lower in patients with AAV than in the controls (*p* < 0.001, *p* = 0.009, *p* < 0.001, and *p* = 0.006, respectively).

**Figure 2:**
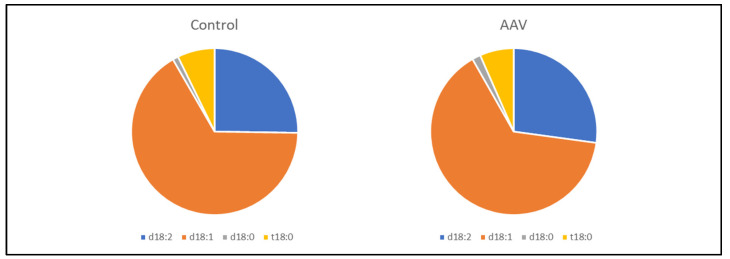
Comparison of the components of serum sulfatides between control and patients with AAV. The compositions of serum sulfatides (LS-d18:2, d18:1, d18:0, and t18:0) were similar between control and AAV patients.

Serum sulfatide levels and the levels of their components, LS-d18:2, d18:1, and t18:0, in patients with AAV were significantly lower than those in control patients (*p* < 0.001, *p* = 0.009, *p* < 0.001, and *p* = 0.006, respectively, Figure 1, Table 1). The composition of serum sulfatides (LS-d18:2, d18:1, d18:0, and t18:0) was similar between controls and AAV patients (Figure 2). Background data of patients with AAV and controls are presented in Table 1. Age, blood pressure, blood urea nitrogen, creatinine, C-reactive protein, soluble thrombomodulin, pentraxin 3, frequency of hypertension, hematuria, and proteinuria were significantly higher in patients with AAV than in the controls (Table 1). Total protein, albumin level, eGFR, total cholesterol, high-density lipoprotein cholesterol, low-density lipoprotein cholesterol, and hemoglobin were significantly lower in patients with AAV than in the controls (Table 1).

### 3.2. Comparison of Clinical Parameters between the Histopathologic Classes of ANCA-Associated Glomerulonephritis

**Figure 3:**
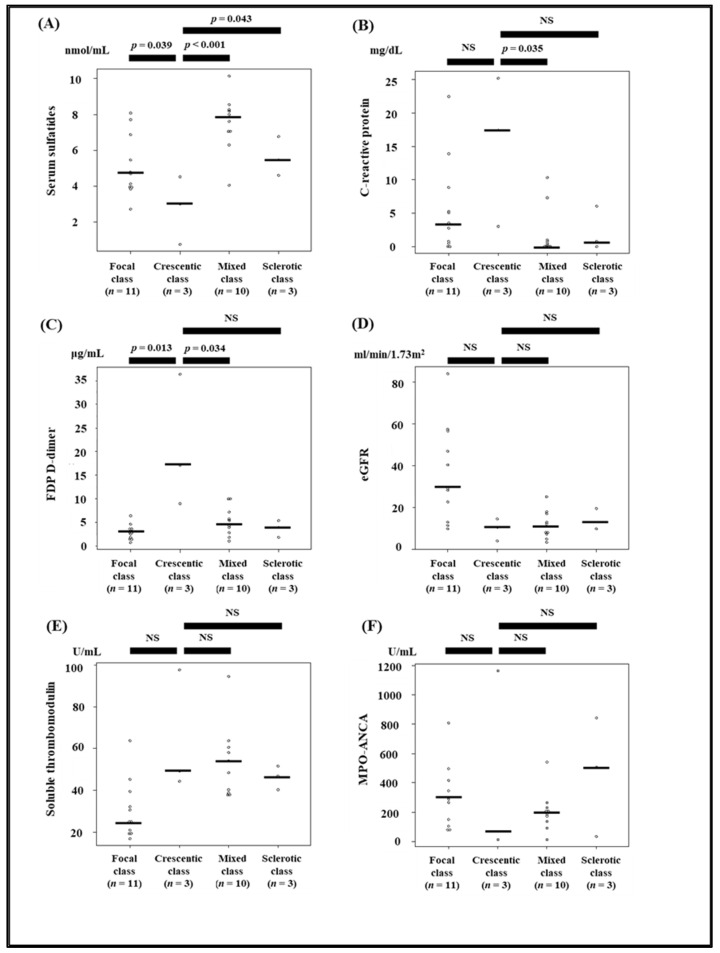
Comparison between candidates of possible AAV disease activity markers, such as serum sulfatides, C-reactive protein, FDP D-dimer, eGFR, soluble thrombomodulin, MPO-ANCA and kidney histopathological findings. Patients were divided into the following four classes based on the histopathologic classification of ANCA-associated glomerulonephritis: focal, crescentic, mixed, and sclerotic. (**A**–**C**) Serum sulfatide levels were significantly lower and C-reactive protein and FDP D-dimer levels were significantly higher in the crescentic class than in the other classes. (**D**,**E**) eGFR and soluble thrombomodulin levels in the crescentic class were significantly lower than those in the focal class. However, no significant differences in eGFR and soluble thrombomodulin were detected between the crescentic class and the mixed or sclerotic classes. (**F**) No significant differences in MPO-ANCA titer were detected between the crescentic class and other classes.

**Figure 4:**
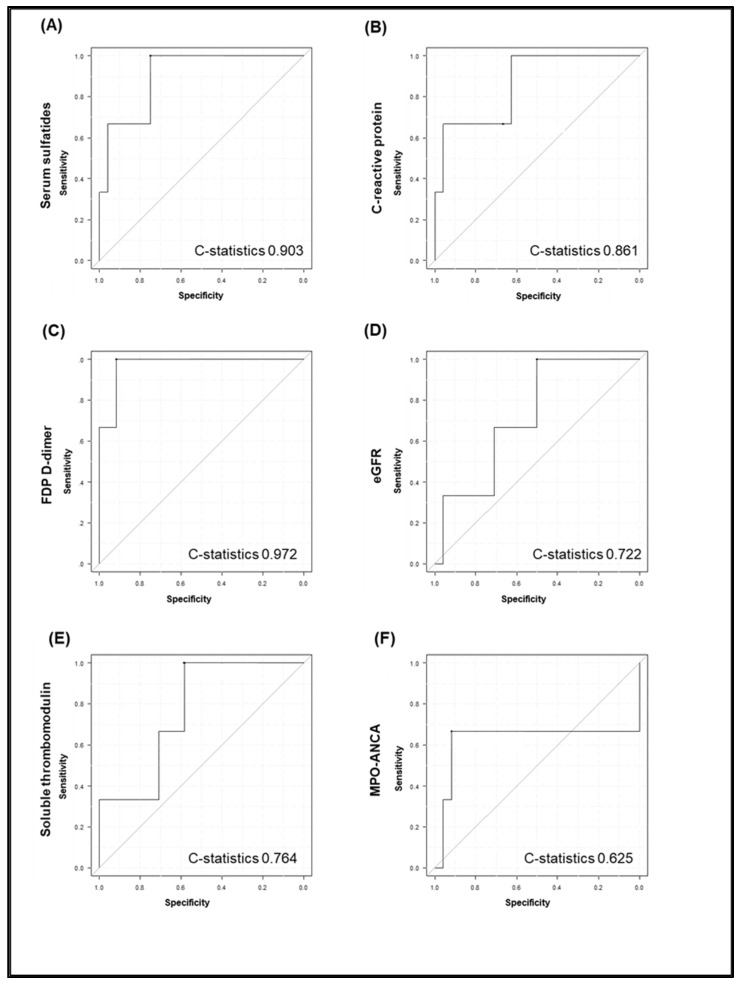
C-statistics that calculated the ability to predict crescentic class lesions and candidates of possible AAV disease activity markers, such as serum sulfatides, C-reactive protein, FDP D-dimer, eGFR, soluble thrombomodulin, and MPO-ANCA titer. C-statistics (predicting crescentic class lesions) of serum sulfatides, C-reactive protein, FDP D-dimer, eGFR, soluble thrombomodulin, and MPO-ANCA titer were 0.903, 0.861, 0.972, 0.722, 0.764, 0.625, respectively (**A**–**F**).

Serum sulfatides, eGFR, FDP D-dimer, soluble thrombomodulin, and hemoglobin levels were significantly different between the four histopathologic classes of ANCA-associated glomerulonephritis (Table 2). Serum sulfatide levels, but not measures of other possible AAV markers, were significantly lower in the crescentic class than those in other classes (Figure 3). C-statistics used to calculate the relationship between the ability to predict crescentic class lesions and candidates of possible AAV disease activity markers, such as serum sulfatides, C-reactive protein, FDP D-dimer, eGFR, soluble thrombomodulin, and MPO-ANCA titer, are presented in Figure 4. The C-statistic of serum sulfatides was high at 0.903; however, it did not significantly differ from that of other markers (*p* = 0.76, *p* = 0.45, *p* = 0.14, *p* = 0.33, and *p* = 0.25 for C-reactive protein, FDP D-dimer, eGFR, soluble thrombomodulin, and MPO-ANCA titer, respectively).

### 3.3. Comparison between Serum Sulfatides, C-Reactive Protein, FDP D-Dimer, eGFR, and Soluble Thrombomodulin, MPO-ANCA and Kidney Histopathological Findings

FDP D-dimer, eGFR, and soluble thrombomodulin levels were significantly correlated with the percentage of active glomerular crescents (Table 3). eGFR and soluble thrombomodulin levels were significantly associated with IFTA > 25%, while serum sulfatides, C-reactive protein, FDP D-dimer, MPO-ANCA levels were not (Table 4).

### 3.4. Correlation between Serum Sulfatide Levels and Clinical Parameters

Serum sulfatide levels were significantly negatively correlated with C-reactive protein and pentraxin 3 (r = −0.713, and r = −0.460, respectively) (Table 5). In addition, serum sulfatide levels were significantly positively correlated with albumin, total cholesterol, high-density lipoprotein cholesterol, and low-density lipoprotein cholesterol levels (r = 0.510, r = 0.722, r = 0.559, and r = 0.527, respectively) (Table 5). There was no significant negative/positive correlation between serum sulfatide levels and MPO-ANCA titers.

### 3.5. Association between Serum Sulfatide Level and Clinical Outcomes/Complications

The median duration of observation of patients in the current study patients was 16 (3–43) months. Clinical outcomes such as all-cause death, end-stage kidney disease, alveolar hemorrhage, interstitial lung lesion, nerve disorder, and thrombotic complications were not significantly associated with serum sulfatide levels (Table 6).

## 4. Discussion

Serum sulfatide levels were lower in patients with AAV than in the controls. The differences in serum sulfatide levels between controls and AAV patients were considered to be a result of serum sulfatide levels because the components of serum sulfatides were similar between controls and AAV patients. Serum sulfatide levels were significantly different between the four histopathologic classes of ANCA-associated glomerulonephritis, and serum sulfatide levels in the crescentic class were significantly lower than those in other classes. In addition, the ability of serum sulfatide levels to predict crescentic class lesions was high, as evaluated by the C-statistics. Serum sulfatide levels significantly correlated with C-reactive protein and pentraxin 3, and serum sulfatide levels may reflect inflammation, particularly in the vascular endothelium. However, serum sulfatide levels were not significantly associated with MPO-ANCA titer.

According to previous studies, sulfatides are produced in the liver and secreted with cholesterol [9,13]. According to basic research using 5/6 nephrectomy mice, it is possible that chronic kidney dysfunction increases uremic toxins such as indoxyl sulfate, resulting in degradation of sulfatides in liver tissue and decreased serum sulfatide levels [10]. Another study suggested that changes in serum sulfatide levels were strongly associated with hepatic expression of cerebroside sulfotransferase, the sulfatide synthesis enzyme [9,10,11,36]. In addition, the hepatic expression of cerebroside sulfotransferase was decreased via hypertension and oxidative stress-related mechanisms, resulting in decreased serum sulfatide levels [11]. The very low serum sulfatide levels in patients with end-stage kidney disease were increased after kidney transplantation [37,38]. Because malondialdehyde, an oxidative stress marker, changes and decreases after kidney transplantation, it is possible that oxidative stress is significantly associated with factors responsible for decreasing serum sulfatide levels [37,38]. Similarly, a decrease in serum sulfatide levels in hemodialysis patients is associated with increased malondialdehyde levels [39]. It is believed that uremic toxins such as indoxyl sulfate and oxidative stress are higher in patients with AAV than in those without AAV because patients who developed AAV presented with kidney dysfunction and a high level of systemic inflammation. Thus, these mechanisms may be associated with lower serum sulfatide levels in patients with AAV than in controls. In addition, although the mechanism of low serum sulfatide levels in patients with AAV categorized as the crescentic class is unknown, one hypothesis is that platelets are activated in severe vasculitis lesions such as necrotizing glomerulonephritis, thereby overexpressing P-selectin, which serum sulfatide may bind to. A previous study indicated that sulfatides bind to P-selectin [12,13]. This leads to consumption and a decrease in the level of serum sulfatides in the lesion. To clarify these hypotheses, we need to investigate the localization of sulfatides in active glomerular lesions.

There have been various studies exploring biomarkers of disease activity in patients with AAV, and biomarkers in clinical practice are ANCA, C-reactive protein, proteinuria, and hematuria [2,7]. ANCA itself contributes to the diagnosis of AAV, and changes in ANCA status or titer may correlate with the disease activity of AAV and risk of relapse [2,7]. However, ANCA titers do not always reflect disease activity in real time, and caution should be exercised when interpreting the status or titer of ANCA [7]. C-reactive protein level reflects the severity of systemic inflammation and is a sensitive disease marker of AAV [2]. However, elevated levels of C-reactive protein are often observed not only in AAV but also in infectious diseases, malignant diseases, and other immunological disorders [8]. Several biomarkers have been identified at the research stage, including E-selectin, P-selectin, intracellular adhesion molecule (ICAM)-3, thrombomodulin, pentraxin 3, matrix metalloproteinase (MMP) 1, MMP3, MMP9, vascular endothelial growth factor (VEGF), tissue inhibitor of metalloproteinase I, chemokine C-X-C motif ligand 13, interleukin 6, semaphorin 4D, and neutrophil extracellular traps [2,7,40,41]. In addition, the following urinary biomarkers were investigated: proteinuria, hematuria, monocyte chemoattractant protein-1, sCD163, and sCD25 [2,7,40]. Most of these markers were associated with adhesion molecules, markers of vascular endothelial function and injury, inflammatory cytokines, and complement related products. Serum sulfatides were significantly correlated with inflammation and intravascular coagulation. Therefore, the combined use of serum sulfatide levels and these markers may be useful for evaluating the severity of AAV. In addition, active crescentic lesions in patients with IgA nephropathy or anti-glomerular basement membrane diseases that cause necrotizing crescent glomerulonephritis may also be associated with alterations in serum sulfatide levels. Future investigation of the association between serum sulfatide levels and crescentic nephritis due to IgA nephropathy and anti-glomerular basement membrane disease will be expected in the future.

In the current study, unfortunately, we did not detect a significant association between serum sulfatide levels and clinical outcomes such as all-cause mortality or kidney prognosis. In addition, we did not detect a significant association between serum sulfatide levels and lung, nerve, and coagulation system complications. This was a small-scale pilot study, and the number of clinical events was small; therefore, large-scale prospective studies are required to evaluate the clinical significance of serum sulfatide levels in clinical settings. In this study, we could not investigate anti-sulfatide antibodies and could not present any values related to anti-sulfatide antibodies. Therefore, the relationship between serum sulfatide levels and anti-sulfatide antibodies remains uncertain, and future investigations will be required to clarify the role between those factors.

## 5. Conclusions

We gained new insights into changes in serum sulfatide levels by studying patients with AAV. Our pilot study indicated the following conclusions: Serum sulfatide levels were significantly lower in patients with AAV than in controls. Although no significant association between serum sulfatide levels and clinical outcomes could be detected in the current study, the reduction in serum sulfatide levels was significantly correlated with C-reactive protein and PTX3 elevation that was indicative of systemic inflammation, including in vascular endothelial cells. These inflammatory responses may cause and reflect the presence of active glomerular lesions, such as crescentic class lesions, that lead to poor renal prognosis in patients with AAV. It is possible that a reduction in serum sulfatide levels may be a useful marker for AAV, and large-scale validation studies will be required in the future.

## Figures and Tables

**Table 1:** Comparison of the background data between control and patients with AAV.

	AAV (*n* = 35)		Control (*n* = 10)		*p*-Value
Age (years)	74 ± 11		57 ± 8		* <0.001
Male (*n*,%)	20	57.1	3	30.0	0.17
BMI (kg/m^2^)	22.4 ± 3.7		23.4 ± 3.4		0.43
Systolic BP (mmHg)	143 ± 27		113 ± 8		* 0.002
Diastolic BP (mmHg)	80 ± 14		70 ± 8		* 0.041
Heart rate (beats/min)	75 ± 14		71 ± 9		0.38
Diabetes mellitus (*n*,%)	10	28.6	2	20.0	0.71
Hypertension (*n*,%)	24	68.6	1	10.0	* 0.002
Smoking history (*n*,%)	20	54.3	4	40.0	0.49
Malignancy (*n*,%)	8	22.9	0	0.0	0.17
**Laboratory data**					
Total protein (g/dL)	6.3 ± 0.8		7.1 ± 0.4		* 0.005
Albumin (g/dL)	2.8 ± 0.7		4.3 ± 0.3		* <0.001
BUN (mg/dL)	47.4 (31.3–75.4)		14.5 (13.5–16.8)		* <0.001
Cre (mg/dL)	3.4 (2.0–5.1)		0.7 (0.6–0.9)		* <0.001
eGFR (mL/min/1.73 m^2^)	12.7 (8.1–23.7)		72.3 (76.6)		* <0.001
CRP (mg/dL)	2.89 (0.38–8.18)		0.04 (0.02–0.11)		* <0.001
Total cholesterol (mg/dL)	176 ± 36		222 ± 39		* 0.001
HDL-C (mg/dL)	40 ± 15		61 ± 18		* 0.001
LDL-C (mg/dL)	103 ± 28		127 ± 28		* 0.023
Triglycerides (mg/dL)	120 (100–167)		153 (81–183)		0.69
Soluble TM (U/mL)	44.1 (35.2–56.6)		13.8 (11.6–15.9)		* <0.001
Pentraxin3 (ng/mL)	6.8 (5.6–39.1)		1.6 (1.2–2.2)		* <0.001
Serum sulfatides (nmol/mL)	5.44 ± 2.14		8.53 ± 1.28		* <0.001
Lysosulfatides-d18:2 (nmol/mL)	1.52 ± 0.71		2.16 ± 0.44		* 0.009
Lysosulfatides-d18:1 (nmol/mL)	3.47 ± 1.31		5.67 ± 0.91		* <0.001
Lysosulfatides-t18:0 (nmol/mL)	0.38 ± 0.23		0.61 ± 0.16		* 0.006
White blood cell count (/µL)	6800 (5650–10,890)		5480 (4332–7462)		0.10
Hemoglobin (g/dL)	9.7 ± 1.6		14.1 ± 1.2		*<0.001
Platelet count (×10^4^/µL)	29.9 ± 12.2		26.8 ± 5.3		0.45
Hematuria (*n*,%)	34	97.1	1	10.0	* <0.001
Proteinuria (*n*,%)	32	91.4	0	0.0	* <0.001
**Clinical events**					
ESKD (*n*,%)	12	34.3	-	-	-
All-cause death (*n*,%)	2	5.7	-	-	-
**AAV-related data**					
MPO-ANCA (*n*,%)	35	100	-	-	-
PR3-ANCA (*n*,%)	0	0	-	-	-
MPO-ANCA titer (U/mL)	316 ± 326		-	-	-
Interstitial lung lesion (*n*,%)	19	54.3	-	-	-
Alveolar hemorrhage (*n*,%)	6	17.1	-	-	-
Neurological disorder (*n*,%)	4	11.4	-	-	-
BVAS	18	11–26	-	-	-
**Treatment pattern**					
PSL (maximum) (mg/kg/day)	0.71		-	-	-
mPSL pulse (*n*,%)	28	80.0	-	-	-
CY (*n*,%)	7	20.0	-	-	-
Rituximab (*n*,%)	6	17.1	-	-	-
Plasma exchange (*n*,%)	7	20.0	-	-	-
TMP-SMX (*n*,%)	34	97.1	-	-	-

The continuous variables exhibiting a normal distribution are presented as means and standard deviations, whereas those exhibiting a non-normal distribution are presented as medians and interquartile ranges. Categorical variables are presented as numbers (*n*) and percentages (%). Comparisons of continuous variables between two groups were conducted by Student’s *t*-test or Mann–Whitney U test for variables with a normal or non-normal distribution, respectively. The comparison of categorical variables was performed using Fisher’s exact probability test. A *p*-value < 0.05 was considered statistically significant (represented with an asterisk *). AAV: ANCA-associated vasculitis, ANCA: anti-neutrophil cytoplasmic antibody, BMI: body mass index, BP: blood pressure, BUN: blood urea nitrogen, BVAS: Birmingham Vasculitis Activity Score, Cre: creatinine, CRP: C-reactive protein, CY: cyclophosphamide, ESRD: end-stage renal disease, eGFR: estimated glomerular filtration rate, HDL-C: high density lipoprotein cholesterol, LDL-C: low density lipoprotein cholesterol, MPO: myeloperoxidase, mPSL: methylprednisolone, PR3: proteinase 3, PSL: prednisolone, RIT: rituximab, Soluble TM: soluble thrombomodulin, TMP-SMX: trimethoprim-sulfamethoxazole.

**Table 2:** Comparison of clinical parameters between the histopathologic classes of ANCA-associated glomerulonephritis.

	Histopathologic Classification of ANCA-Associated Glomerulonephritis								
	Focal		Crescentic		Mixed		Sclerotic		*p*-Value
	*n* = 11		*n* = 3		*n* = 10		*n* = 3		
Age (years)	73 ± 7		62 ± 16		71 ± 10		71 ± 16		0.51
Male (*n*,%)	6	54.5	1	33.3	6	60.0	1	33.3	0.84
BMI (kg/m^2^)	22.3 (20.4–23.6)		22.6 (22.6–24.8)		22.5 (20.4–23.7)		19.9 (19.4–25.2)		0.76
Systolic BP (mmHg)	129 ± 25		136 ± 26		159 ± 30		142 ± 27		0.13
Diastolic BP (mmHg)	73 ± 11		81 ± 19		88 ± 16		82 ± 12		0.13
Heart rate (/min)	73 ± 12		96 ± 17		74 ± 11		78 ± 15		0.06
Diabetes mellitus (*n*,%)	5	45.4	0	0	3	30.0	0	0	0.36
Hypertension (*n*,%)	8	72.7	1	33.3	7	70.0	2	66.7	0.73
Smoking history (*n*,%)	6	54.5	1	33.3	7	70.0	1	33.3	0.63
Malignancy (*n*,%)	1	9.1	1	33.3	3	30.0	0	0	0.40
**Laboratory data**									
Total protein (g/dL)	6.6 ± 0.8		6.1 ± 0.4		6.3 ± 0.5		7.4 ± 0.3		0.06
Albumin (g/dL)	2.9 ± 0.5		2.0 ± 1.0		3.1 ± 0.6		3.1 ± 0.7		0.12
eGFR (mL/min/1.73 m^2^)	29.2 (17.6–51.8)		10.9 (7.3–12.8)		10.1 (7.4–15.7)		13.3 (11.6–16.4)		* 0.016
CRP (mg/dL)	3.4 (0.6–7.0)		17.3 (10.2–21.3)		0.4 (0.1–0.9)		0.8 (0.5–3.4)		0.11
Soluble TM (U/mL)	25.3 (20.1–35.9)		49.4 (46.8–73.6)		51.2 (39.1–59.6)		47.2 (43.6–49.6)		* 0.014
Bold is Pentraxin3 (ng/mL)	6.8 (2.9–22.0)		55.4 (46.8–76.5)		6.4 (5.3–10.2)		5.0 (5.0–22.5)		0.14
Serum sulfatides (nmol/mL)	5.1 ± 1.7		2.7 ± 1.9		7.5 ± 1.6		5.6 ± 1.1		* <0.001
White blood cell count (/µL)	8790 (6310–14,450)		4828 (2928–7749)		6400 (5862–8430)		5810 (4455–6855)		0.23
Hemoglobin (g/dL)	10.8 ± 1.4		9.1 ± 2.0		9.6 ± 1.3		8.1 ± 0.4		* 0.028
Platelet count (×10^4^/µL)	36.3 ± 12.2		30.4 ± 2.8		25.6 ± 7.4		41.2 ± 15.9		0.07
FDP D-dimer (µg/mL)	2.9 (1.7–3.5)		17.2 (13.1–26.8)		4.9 (3.1–6.7)		3.8 (2.8–4.5)		* 0.017
Hematuria (*n*,%)	11	100	3	100	9	90.0	3	100	0.59
Proteinuria (*n*,%)	11	100	3	100	10	100	3	100	-
**Clinical events**									
ESKD (*n*,%)	1	9.1	2	66.7	3	30.0	0	0	0.12
All cause death (*n*,%)	0	0	0	0	1	10.0	0	0	0.59
**AAV-related data**									
MPO-ANCA (*n*,%)	11	100	3	100	10	100	3	100	-
PR3-ANCA (*n*,%)	0	0	0	0	0	0	0	0	-
MPO-ANCA titer (U/mL)	289 (128–383)		72 (41–616)		194 (145–229)		507 (268–674)		0.60
Interstitial lung lesion (*n*,%)	6	54.5	3	100	5	50.0	1	33.3	0.50
Alveolar hemorrhage (*n*,%)	2	18.2	1	33.3	1	10.0	0	0	0.86
Neurological disorder (*n*,%)	2	18.2	0	0	1	10.0	0	0	1.00
BVAS	18 ± 4		21 ± 4		18 ± 2		16 ± 4		0.33
**Treatment pattern**									
PSL (maximum) (mg/kg/day)	0.74 ± 0.11		0.63 ± 0.14		0.75 ± 0.15		0.70 ± 0.14		0.57
mPSL pulse (*n*,%)	9	81.8	3	100	10	100	2	66.7	0.42
CY (*n*,%)	3	27.3	0	0	2	20.0	2	66.7	0.43
Rituximab (*n*,%)	1	9.1	0	0	3	30.0	1	33.3	0.40
Plasma exchange (*n*,%)	2	18.2	1	33.3	2	20.0	0	0	1.00
TMP-SMX (*n*,%)	11	100	3	100	10	100	3	100	-

The continuous variables exhibiting a normal distribution are presented as means and standard deviations, and those exhibiting a non-normal distribution are presented as medians and interquartile ranges. Categorical variables are presented as numbers (*n*) and percentages (%). Clinical parameters were compared between the four histopathologic classes of ANCA-associated glomerulonephritis by one-way ANOVA or a Kruskal–Wallis test for variables with a normal or non-normal distribution, respectively. The comparison of categorical variables was performed by chi-square test. A *p*-value < 0.05 was considered statistically significant (represented with an asterisk *). AAV: ANCA-associated vasculitis, ANCA: anti-neutrophil cytoplasmic antibody, BMI: body mass index, BP: blood pressure, BUN: blood urea nitrogen, BVAS: Birmingham Vasculitis Activity Score, Cre: creatinine, CRP: C-reactive protein, CY: cyclophosphamide, ESRD: end-stage renal disease, eGFR: estimated glomerular filtration rate, FDP D-dimer: fibrin/fibrinogen degradation products (FDP) D-dimer, HDL-C: high density lipoprotein cholesterol, LDL-C: low density lipoprotein cholesterol, MPO: myeloperoxidase, mPSL: methylprednisolone, PR3: proteinase 3, PSL: prednisolone, RIT: rituximab, Soluble TM: soluble thrombomodulin, TMP-SMX: trimethoprim- sulfamethoxazole.

**Table 3:** Correlation between possible AAV markers (serum sulfatides, C-reactive protein, FDP D-dimer, eGFR, soluble thrombomodulin and MPO-ANCA) and glomerular lesions.

	r	*p* Value
Serum sulfatides		
Active glomerular crescent (%)	−0.265	0.18
Global sclerosis (%)	0.151	0.45
C-reactive protein		
Active glomerular crescent (%)	0.161	0.42
Global sclerosis (%)	−0.272	0.17
FDP D-dimer		
Active glomerular crescent (%)	0.641	* <0.001
Global sclerosis (%)	−0.008	0.97
eGFR		
Active glomerular crescent (%)	−0.549	* 0.003
Global sclerosis (%)	−0.473	* 0.013
Soluble thrombomodulin		
Active glomerular crescent (%)	0.583	* 0.001
Global sclerosis (%)	0.327	0.10
MPO-ANCA titer		
Active glomerular crescent (%)	0.080	0.69
Global sclerosis (%)	−0.072	0.72

A *p*-value < 0.05 was considered statistically significant (represented with an asterisk *). ANCA: anti-neutrophil cytoplasmic antibody, eGFR: estimated glomerular filtration rate, FDP D-dimer: fibrin/fibrinogen degradation products (FDP) D-dimer, MPO: myeloperoxidase.

**Table 4:** Association between possible AAV markers (serum sulfatides, C-reactive protein, FDP D-dimer, eGFR, soluble thrombomodulin and MPO-ANCA) and interstitial fibrosis and tubular atrophy.

	Odds Ratio	95% Confidence Interval	*p*-Value
Serum sulfatides	1.03	0.14–11.1	0.88
C-reactive protein	1.01	0.90–1.12	0.92
FDP D-dimer	0.99	0.89–1.11	0.91
eGFR	0.91	0.83–0.99	* 0.028
Soluble thrombomodulin	1.07	1.00–1.14	* 0.041
MPO-ANCA titer	1.00	0.99–1.01	0.13

A *p*-value < 0.05 was considered statistically significant (represented with an asterisk *). ANCA: anti-neutrophil cytoplasmic antibody, eGFR: estimated glomerular filtration rate, FDP D-dimer: fibrin/fibrinogen degradation products (FDP) D-dimer, MPO: myeloperoxidase.

**Table 5:** Correlation between clinical parameters and serum sulfatide levels.

	Serum Sulfatide Levels	
	r	*p* Value
Age (years)	−0.036	0.84
BMI (kg/m^2^)	0.006	0.98
Systolic BP (mmHg)	0.296	0.08
Diastolic BP (mmHg)	0.143	0.41
Heart rate (beats/min)	−0.197	0.26
BVAS	−0.102	0.56
Alb (g/dL)	0.510	* 0.002
eGFR (mL/min/1.73 m^2^)	0.011	0.95
C-reactive protein (mg/dL)	−0.713	* <0.001
White blood cell count (/µL)	−0.208	0.23
Hemoglobin (g/dL)	0.111	0.53
Platelet count (×10^4^/µL)	−0.036	0.84
Total cholesterol (mg/dL)	0.722	* <0.001
HDL-C (mg/dL)	0.559	* <0.001
LDL-C (mg/dL)	0.527	* 0.001
Triglyceride (mg/dL)	0.064	0.71
Fibrinogen (mg/dL)	−0.098	0.58
FDP-D dimer (μg/mL)	−0.306	0.07
Soluble thrombomodulin (U/mL)	0.052	0.76
Pentraxin3 (ng/mL)	−0.460	* 0.005
MPO-ANCA titer (U/mL)	0.039	0.82

Pearson’s rank correlation or Spearman’s rank correlation coefficient was used to perform a correlation analysis for variables with a normal or non-normal distribution, respectively. A *p*-value < 0.05 was considered statistically significant (represented with an asterisk *). Alb: albumin, ANCA: anti-neutrophil cytoplasmic antibody, BMI: body mass index, BP: blood pressure, BVAS: Birmingham vasculitis activity score, eGFR: estimated glomerular filtration rate, FDP D-dimer: fibrin/fibrinogen degradation products (FDP) D-dimer, HDL-C: high density lipoprotein cholesterol, LDL-C: low density lipoprotein cholesterol, MPO: myeloperoxidase.

**Table 6:** Association between serum sulfatide levels and clinical outcomes/complications.

	Odds Ratio	95% Confidence Interval	*p*-Value
Death	1.02	0.52–2.00	0.96
End stage kidney diseases	1.07	0.74–1.56	0.71
Alveolar hemorrhage	1.09	0.72–1.66	0.67
Interstitial lung lesion	0.91	0.66–1.25	0.56
Nerve disorder	0.73	0.42–1.28	0.28
Thrombotic complications	0.88	0.49–1.56	0.66

A *p*-value < 0.05 was considered statistically significant (represented with an asterisk *).

## Data Availability

The data used in this study are available from the corresponding author upon request.
